# Femoropopliteal Bypasses with Varicose Great Saphenous Vein

**DOI:** 10.3400/avd.cr.21-00120

**Published:** 2022-06-25

**Authors:** Sosei Kuma, Kazuomi Iwasa, Takuya Matsumoto, Hideaki Uchiyama

**Affiliations:** 1 Department of Vascular Surgery, Kyushu Central Hospital, Fukuoka, Fukuoka, Japan; 2 Department of Vascular Surgery, National Hospital Organization Fukuoka-Higashi Medical Center, Koga, Fukuoka, Japan; 3 Department of Surgery, National Hospital Organization Fukuoka-Higashi Medical Center, Koga, Fukuoka, Japan

**Keywords:** peripheral arterial disease, varicose vein, long saphenous vein

## Abstract

The great saphenous vein is the conduit of choice for femoropopliteal or infrapopliteal bypass, but it is traditionally recommended that varicose vein grafts (VVGs) should not be used for bypass conduits owing to the risk of immediate rupture or long-term aneurysmal change. Herein, we report two cases of femoropopliteal bypass with VVGs. They achieved primary patency without aneurysmal formation after 32 and 17 months. Therefore, VVGs without morphologically conspicuous abnormalities are worth considering for usage as a vein graft.

## Introduction

The great saphenous vein (GSV) is the conduit of choice for femoropopliteal or infrapopliteal bypass,^1)^ but it is traditionally recommended that varicose vein grafts (VVGs) should not be used for bypass conduits owing to the risk of immediate rupture or long-term aneurysmal change.^2,3)^

Herein, we report two cases of femoropopliteal bypass with a VVG.

## Case Report

### Case 1

A 74-year-old woman was admitted to our hospital owing to right calf claudication. She had a history of hypertension and dyslipidemia and had undergone endovascular therapy (EVT) for the right external iliac artery and superficial femoral artery (SFA) 7 years earlier.

A physical examination of the right lower extremity performed on presentation was notable for diminished popliteal arterial pulsation, and the right and left ankle brachial pressure indices (ABI) were 0.74 and 0.88, respectively. Duplex ultrasonography (DUS) revealed the in-stent restenosis of the SFA; thus, balloon angioplasty with a scoring balloon (NSE PTA 6×40 mm; Nipro Co., Osaka, Japan) was performed, which increased the right ABI to 0.92.

**Figure 1:**
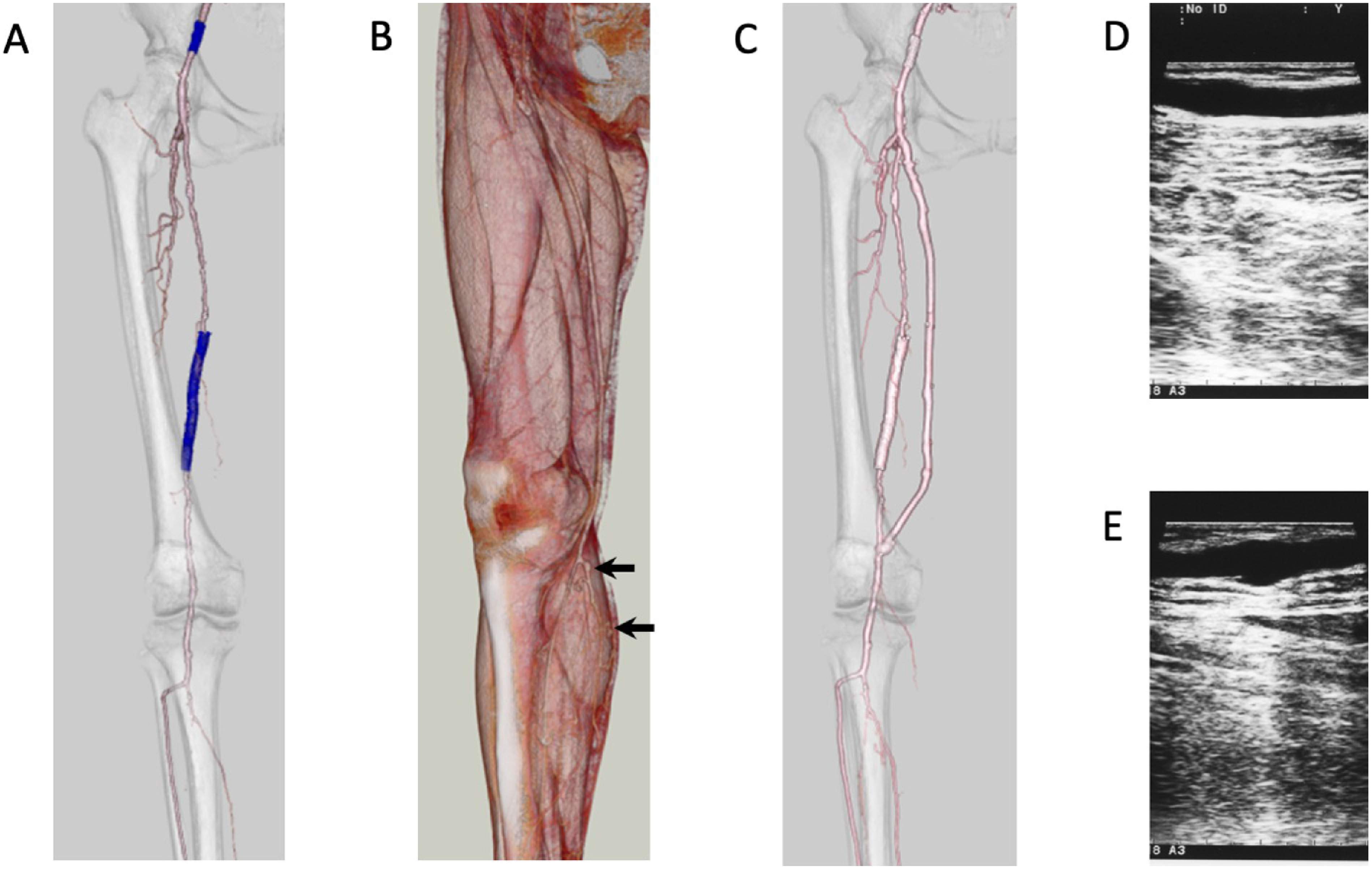
Fig. 1 Case 1. (**A**) Preoperative computed tomography angiography revealed the patency of the right iliac and popliteal arteries as well as stenosis of the right superficial femoral artery stent. (**B**) Preoperative computed tomography (CT) venography revealed the great saphenous vein having no morphologically conspicuous abnormalities except for some segmental type varicosity at the crus (arrows) and some sinusoid dilatations at the origin of valves. (**C**) Postoperative CT angiography revealed that the right femoropopliteal bypass was patent. (**D**) Duplex ultrasonography (DUS) showed no structural changes in the vein graft. (**E**) DUS showed some sinusoid dilatations at the origin of the valves.

One month after this reintervention, the patient was admitted owing to varicosity, edema, and dullness on the right crus. DUS revealed reflux of the saphenofemoral junction (SFJ) but no aneurysmal change in the right GSV. Compression therapy was performed, but no satisfactory improvement was observed in her symptoms. One year after reintervention, the patient complained of right calf claudication again. DUS revealed recurrent restenosis of the SFA stent, and the right ABI dropped to 0.69. Computed tomography angiography (CTA) revealed the patency of the right iliac and popliteal arteries and stenosis of the right SFA stent (**Fig. 1A**). Computed tomography (CT) venography revealed segmental type varicosity at the crus and some sinusoid dilatations at the origin of valves (**Fig. 1B**). We decided to perform femoropopliteal bypass with the varicose GSV owing to her severe claudication, repeated restenosis, and obstinate congestion symptoms.

Surgery was performed under general anesthesia. First, the femoral and above-knee popliteal arteries were exposed and encircled. Then, the GSV was exposed through the same incision, dividing its tributaries. Following systemic heparinization, the SFJ was transected, and the venotomy was closed with continuous sutures (5-0 polypropylene). An arteriotomy was performed for the terminal common femoral artery, and a GSV graft was anastomosed with 6-0 polypropylene continuous sutures as an in situ graft. The distal GSV was dissected and transected, and valvulotomy was performed using a self-sizing expandable valvulotome (LeMaitre Vascular Inc., Burlington, MA, USA). The vein was instantly ligated at the end, and visible tributaries were ligated under angiography. Arteriotomy was performed on the popliteal artery, and the vein graft was anastomosed with 6-0 polypropylene continuous sutures.

ABI increased to 0.94 on the right leg. Her postoperative course was uneventful, and she was discharged home 12 days after the operation. CTA of the lower extremity performed 1 year after the operation demonstrated a widely patent femoropopliteal bypass graft without structural changes (**Fig. 1C**). She has been doing well during the 32-month follow-up period after the operation, and no structural changes have been observed on DUS, although some sinusoid dilatations at the origin of valves have been noted (**Figs. 1D** and **1E**).

### Case 2

**Figure 2:**
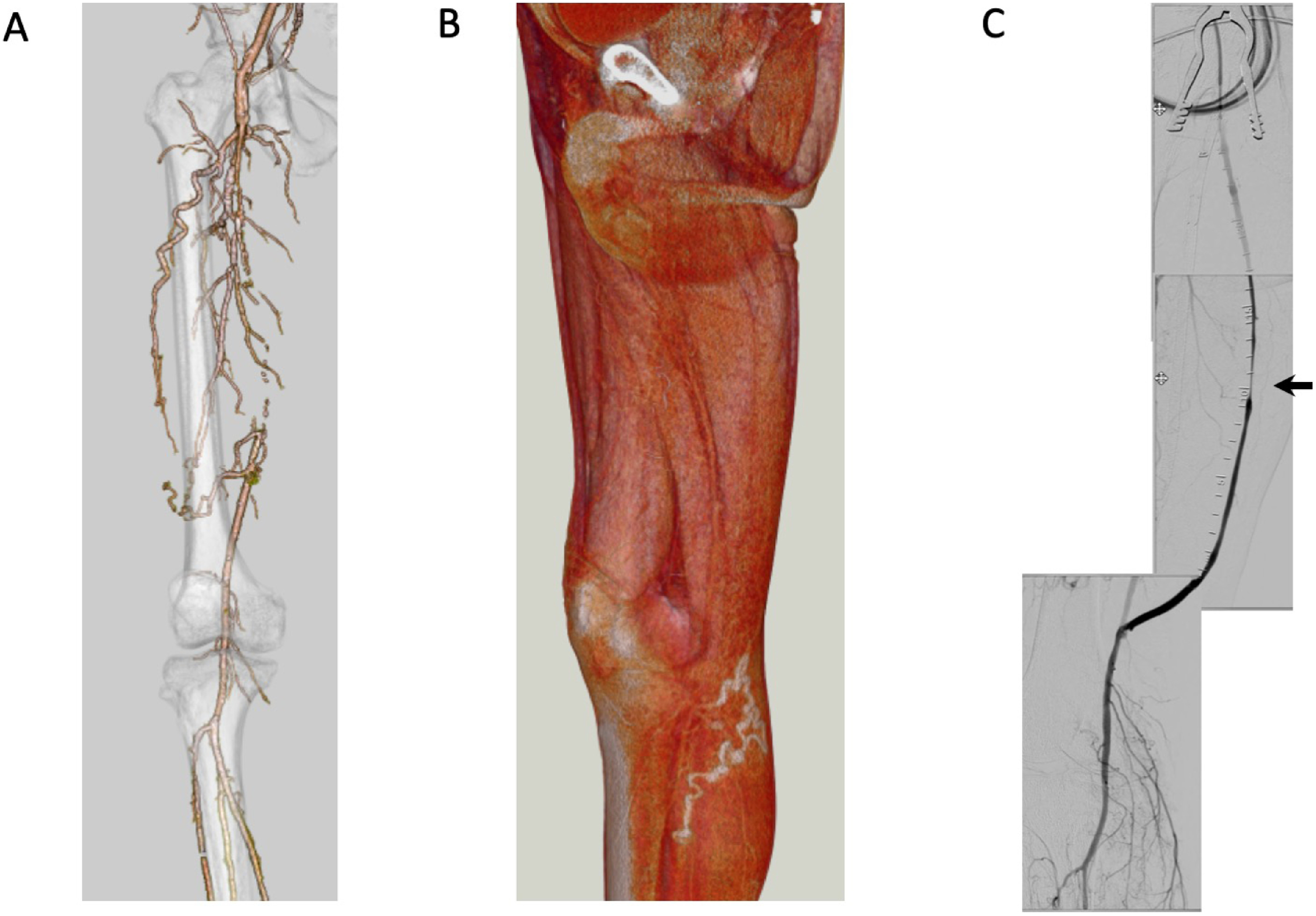
Fig. 2 Case 2. (**A**) Preoperative computed tomography angiography revealed that the superficial femoral artery was occluded. (**B**) Preoperative computed tomography venography revealed the great saphenous vein having no morphologically conspicuous abnormalities except for some segmental type varicosity at the crus and some sinusoid dilatations at the origin of valves. (**C**) Completion angiography revealed that the right femoropopliteal bypass was patent. Vasospasm was observed in the midportion of the vein graft (arrows).

An 89-year-old man was admitted to our hospital owing to pain at rest in the right forefoot. He had a history of hypertension, diabetes mellitus, chronic kidney disease (CKD) stage 3, and chronic heart failure. A physical examination of his right lower extremity performed on presentation was notable for diminished popliteal arterial pulsation. The right and left ABIs were 0.52 and 0.46, respectively. CTA revealed occlusion of the right SFA (**Fig. 2A**); thus, EVT was performed using self-expandable nitinol stents (Innova 6×180 mm and 6×60 mm; Boston Scientific Co., Marlborough, MA, USA).

Three months after the intervention, the patient was admitted to our hospital owing to dullness, eczema, and pigmentation on the left crus. DUS revealed the reflux of the left sapheno–popliteal junction and right SFJ junction. CT venography revealed that the GSV had no morphologically conspicuous abnormalities except for some segmental type varicosity at the crus and some sinusoid dilatations at the origin of valves (**Fig. 2B**). He underwent endovenous laser ablation (EVLA) of the left lesser saphenous vein. Seven months after the intervention, DUS revealed stent occlusion, and the right ABI had dropped to 0.51. Reintervention was not performed due to lack of ischemic complaints and CKD progression. However, he was readmitted owing to the pain of the right forefoot at rest 3 months after stent occlusion, and the right ABI decreased to 0.39. Therefore, we decided to perform femoropopliteal bypass with a varicose GSV due to chronic limb-threatening ischemia (CLTI) and CKD stage 4.

Surgery was performed in a manner similar to that in Case 1. Completion angiography showed that the right femoropopliteal bypass was patent, and vasospasm was also observed in the midportion of the vein graft (**Fig. 2C**). Although he required treatment with diuretics for congestive heart failure postoperatively, he was discharged home 42 days after the operation. ABI increased to 1.22 on the right leg. Neither CTA nor angiography of the lower extremity was conducted owing to CKD, but DUS performed 15 months after the surgery demonstrated widely patent femoropopliteal bypass graft with no structural changes. Although he died from acute exacerbation of chronic heart failure 17 months after the operation, the primary patency of the bypass graft and limb salvage was successfully achieved for the remainder of his life.

## Discussion

The choice of graft material is important in cases of infrainguinal bypass to ensure the maintenance of graft patency. The European Society of Cardiology guideline 2019 on peripheral arterial disease recommends the GSV as the conduit of choice for femoropopliteal or infrapopliteal bypass.^1)^ However, graft failure can occur even with autologous vein grafts. Neointimal hyperplasia, which can induce graft stenosis or occlusion, is a major cause of graft failure, and vein graft aneurysm (VGA) is a rare complication, occurring in 1.8%–3.8% of cases.^4)^ Traditionally, it is recommended that VVGs should not be used for bypass conduits owing to the risk of immediate rupture or long-term aneurysmal change.^2,3)^

We described two cases wherein femoropopliteal bypass with varicose GSV was performed. Although EVT was suitable for use in the revascularization of Case 1, it carried a risk of causing further restenosis. Additionally, the patient hoped to have her varicose veins treated due to insufficient improvement with compression therapy. Although VGA formation was concerning owing to her long life expectancy, long-term patency could be expected; thus, a femoropopliteal bypass with a GSV graft was performed to treat her varicose great saphenous vein. Because of his CLTI status due to reocclusion, Case 2 required revascularization of the right leg. Although EVT might have been suitable for revascularization, reocclusion was expected to result in poor patency.^5)^ Furthermore, the progressive nature of his CKD made us hesitant to perform EVT, which would require more contrast. Although he was elderly with various comorbidities, a femoropopliteal bypass with a GSV graft was performed.

**Figure 3:**
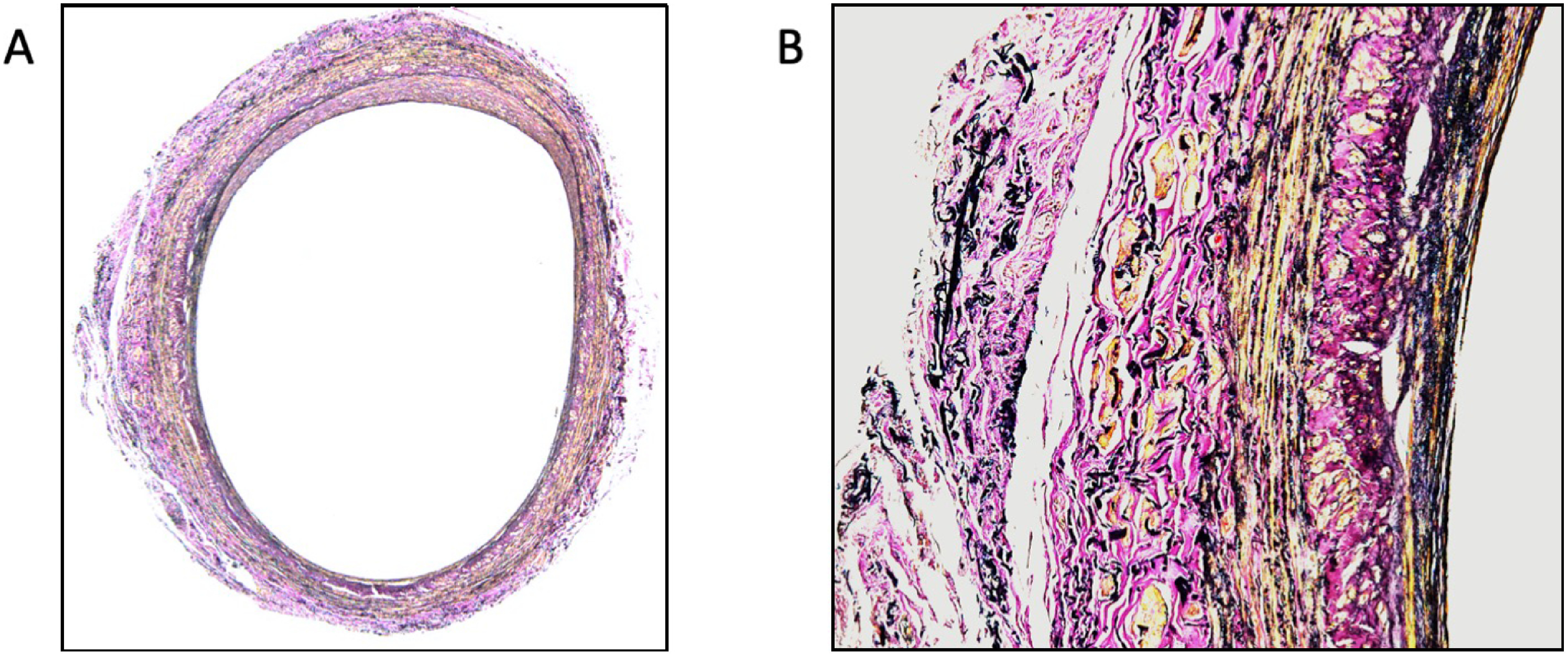
Fig. 3 Histopathology of varicose veins obtained from a patient other than Cases 1 or 2. There was no muscle layer thinning or disappearance of elastic fibers, although there was intimal hyperplasia and collagen deposition at the intima and adventitia (**A**, original magnification ×40; **B**, original magnification ×100).

Histopathologically or physiologically, varicose veins show intimal hyperplasia, collagen deposition, fragmentation or disappearance of elastic fibers, and reduced vascular reactivities.^6,7)^ These findings make it easy to imagine that VVG is prone to VGA. Although infrapopliteal bypass may allow the use of VVG in favor of patency over the risk of VGA formation,^8,9)^ a prosthetic graft is an acceptable alternative for above-knee femoropopliteal bypass if a GSV is not available.^1)^ However, the degree and extent of these changes, which appear as dilatation, aneurysm, or tortuosity, vary among patients, and it is unclear whether vein grafts with minimal or mild changes develop aneurysmal degeneration. Indeed, the genicular portion of the GSV specimen, which was obtained during the other’s EVLA, showed no muscle layer thinning or disappearance of elastic fibers, although intimal hyperplasia and collagen deposition were noted in the intima and adventitia (**Fig. 3**). Furthermore, the vasospasm of the vein graft observed on completion angiography of Case 2 might reflect the fact that its vascular reactivity was retained to a certain extent (**Fig. 2C**). Although the indications for VVG should be determined in individual cases with reference to the degree or extent of varicosity changes, the site of distal anastomosis, the patency of the distal runoff vessels, and life expectancy, we speculate that at least the usage of VVGs without morphologically conspicuous abnormalities could be permissible. Although some may hold the opinion that VVGs should not be used as in situ vein grafts, as whole visual observation cannot be performed or a valvulotome may injure a vessel wall,^9)^ in these two cases, a VVG was used as in situ vein graft for femoropopliteal bypass because there were no morphologically conspicuous abnormalities. In previous studies, minor dilatations of varicose veins were corrected using plication, tuck stitching, resection with anastomosis, or prosthetic reinforcement. However, our cases did not require these additional procedures.

Presently, evidence concerning the long-term outcomes of VVGs is insufficient. The anecdotal case reports of VVG application reported midterm results regarding the patency and aneurysmal changes.^8,10)^ Kim et al. reported a case of infragenicular bypass using a VVG for acute limb ischemia during 72-month follow-up without recurrent symptoms or revision.^9)^ Recently, Neufang et al. reported good late graft patency and limb salvage (54% primary patency and 83% limb salvage rates at 5 years) combined with a low rate of late vein graft degeneration using external polytetrafluoroethylene reinforcement of VVGs.^2)^ Our cases achieved primary patency without aneurysmal formation within 32 and 17 months. However, since VGA usually occurs more than 2 years after bypass, due to progressive atherosclerosis, further follow-up is required.

## Conclusion

Herein, we report two cases of femoropopliteal bypass with a VVG, which achieved good midterm results. Therefore, VVGs without any morphologically conspicuous abnormalities are worth considering for usage as a vein graft.
